# Temperature, precipitation, ozone pollution, and daily fatal unintentional injuries in Jiangsu Province, China during 2015–2017

**DOI:** 10.1186/s40621-020-00268-9

**Published:** 2020-07-27

**Authors:** Leon S. Robertson, Lian Zhou, Kai Chen

**Affiliations:** 1 Yale University School of Public Health, 60 College Street, New Haven, CT 06520-8034 USA; 2 Jiangsu Provincial Center for Disease Prevention and Control, 172 Jiangsu Road, Nanjing, 21009 Jiangsu China; 3 Yale Center on Climate Change and Health, Yale University School of Public Health, 60 College Street, New Haven, CT 06520-8034 USA

**Keywords:** Unintentional injury mortality, Temperature, Air pollution, Avoidance behavior

## Abstract

**Background:**

The correlation of unintentional injury mortality to rising temperatures found in several studies could result from changes in behavior that increases exposure to hazards or risk when exposed. Temperature, precipitation and air pollutants may contribute to symptoms and distractions that increase risk or avoidance behavior that reduces risk. This study examines data that allows estimates of the relation of daily maximum temperature, precipitation and ozone pollution to injury mortality risk, each corrected statistically for the correlation with the others.

**Methods:**

Daily data on unintentional injury deaths and exposures to temperature, precipitation and ozone in 9 cities in Jiangsu Province, China during 2015–2017 were analyzed using Poisson regression. The regression estimates were adjusted for weekends, holidays, an anomalous difference in death rates in Nanjing, and population size.

**Results:**

Non transport injury death risk increased substantially in relation to higher temperatures when temperatures were in the moderate range and even more so at temperatures 35 degrees (C) and higher. Transport deaths were related to increasing deaths when temperatures were low but the correlation reversed at higher temperatures. Deaths were lower on rainy days when temperatures were cool and moderate with the exception of non-transport injuries when temperatures were moderate. Higher ozone concentrations were associated with more deaths except when temperatures were low.

**Conclusions:**

The variations in deaths in relation to temperature, precipitation and ozone suggest that people are behaving differently or are in different environments when specific combinations of the predictor variables are prevalent, putting them at greater or less risk. More study of the behaviors and circumstances that result in injury under those conditions is needed.

## Background

Temperature, precipitation and air pollution may affect risk of injury in two ways: behavior changes that increase or decrease exposure to potentially injurious energy or increased risk if exposed. A review of the literature on the correlation of various types of injuries to ambient temperature noted that most studies focused on the association of injuries with extreme temperatures but those that studied temperature in normal ranges also found increased injury incidence and mortality rates associated with warmer temperatures (Kampe et al. 2016). Comparisons among U.S. states indicate increased injury mortality risk associated with warmth above normal (Parks et al. 2020). Research on police-reported road collisions in Spain found a 2.9% increase during heat waves (Basagana 2015). While peoples’ attention to tasks at hand may be distracted by extreme temperatures, the more likely explanation for much of the correlation is changes in human activity based on temperature and precipitation that expose them to greater or less environmental hazards. Although certain recreational activities, such as skiing, are more frequent on colder days, many others, such as swimming and boating are warm and dry weather activities. Outdoor construction and other projects are sometimes suspended because of inclement weather. Higher temperatures increase risk for cocaine users (Marzuk et al. 1998) but not as high as once thought (Bohnert et al. 2010). The correlation of temperature to drug overdoses could also partly be the result of addicts being more often under the watch of families or other persons who care for them on cold or wet days that inhibit freedom of movement.

A study of road deaths among urban cities and counties in the U.S. found that reversal of the decreasing road death trend during 2015 was mainly associated with increased temperatures in that year (Robertson 2018a). An alternative hypothesis that economic recovery from the Great Recession of 2008–2009 explained the reversal was not confirmed by comparing data among U.S. states during 2000–2016 (Robertson 2018b). In both studies, increases in vehicle travel were found in relation to increasing temperature. Road use by pedestrians and bicyclists likely increased as well. Researchers noting the increase in injury deaths in Chinese cities during the latter half of the twentieth Century repeated without evidence the conventional wisdom that they are mainly the result of activities associated with rapid economic growth (Zhou 2012). From 2010 to 2015, however, injury death rates in Chinese cities were substantially lower than in the 1980s and 1990s while economic growth remained high (Ozanne-Smith and Li 2018).

Environmental conditions other than temperature and precipitation may increase or decrease injury risk to those exposed and may also alter the probability of exposure (Sager 2019). Air pollution is irritating to the eyes and may impair vision as well as lead to coughing spasms and breathing problems especially among the asthmatic, all of which would distract from alertness. Sleepiness has been associated with air pollutants (Heyes and Zhu 2019). Performance on verbal and mathematics tests is reduced among those exposed to higher ranges of air pollutants (Zhang et al. 2018). Attempts to reduce exposure to pollutants may lead to more sedentary activities that lower risk of injury (Bresnahan et al. 1997). Studies of avoidance behavior in relation to pollution find that some people stay indoors when the air has higher concentrations of pollutants, particularly when there are broadcast, print and internet warnings of hazard to health. Smog alerts in California were related to less attendance at studied outdoor venues (Neidell 2008). School absences in Texas were found related to higher concentration of air pollutants (Curry et al. 2009) which could be due to both increased illness and avoidance behavior. Adults with asthma change their activities in response to changes in relatively low levels of ozone in the environment (Eiswerth et al. 2005).

The purpose of this paper is to report analysis of the extent to which daily fluctuations in temperature, precipitation and ozone concentrations were related to the daily counts of fatal injury during 2015 through 2017 in nine cities located in Jiangsu Province, China. While the analysis of injury deaths in correlation with these factors does not specify the extent of avoidance behavior versus risk when exposed, the analysis does quantify the likely net effect of these two factors and, in the instances of negative correlations, suggests that avoidance behavior reduces the overall risk.

## Methods

Daily injury deaths during 2015–2017 in each of 9 cities in Jiangsu Province, China were provided by the Jiangsu Provincial Center for Disease Prevention and Control. The deaths were specified as occurring in transport or other circumstances. This classification was based on the International Statistical Classification of Diseases and Related Health Problems 10th Revision (ICD-10) codes V00-V99 for transport cases and W00-W99 for other unintentional cases (ICD10Data.com 2019). As of 2013, 96.5% of transport deaths in China were from road injuries (Zhou et al. 2016).

Daily meteorological data from each city, including daily maximum, minimum and average temperatures, and precipitation, were obtained from the China Meteorological Data Sharing Service System. Daily city-level air pollution data during 2015–2017 were collected from the National Air Pollution Monitoring System, whose quality was assured by the Ministry of Ecology and Environment of China. A tornado in Yancheng June 23, 2016 killed about 100 people in one day. These were excluded to prevent a skewed death distribution from a rare event. Data on the yearly population of each city was obtained from the Jiangsu Statistical Yearbooks (2015–2017).

Graphs of death rates per billion person days of exposure to each degree of maximum daily temperature were examined for evidence of nonlinearity. Based on the nonlinearity of deaths in relation to temperature, shown in the results section, separate Poisson regression models were fitted for temperatures below 25 degrees, 25–34 degrees and 35+ degrees (C). To reduce the skew of the distributions of precipitation, and ozone (O_3_), the square root of precipitation and the natural logarithm of ozone were used in the analysis.

The form of the regression equation is:

$$ \mathrm{Deaths}=\mathrm{Intercept}+\left({\mathrm{b}}_1\ \mathrm{x}\ \mathrm{temperature}\right)+\left({\mathrm{b}}_2\ \mathrm{x}\surd \mathrm{precipitation}\right)+\left({\mathrm{b}}_3\ \mathrm{x}\ \log\ \left({\mathrm{O}}_3\right)\right)+\left({\mathrm{b}}_4\ \mathrm{x}\ \mathrm{weekend}\right)+\left({\mathrm{b}}_5\ \mathrm{x}\ \mathrm{holiday}\right)+\left({\mathrm{b}}_6\ \mathrm{x}\ \mathrm{Nanjing}\right). $$

where weekend is 1 if a Saturday or Sunday that is not indicated as a workday on the Chinese holiday calendar (timeanddate.com 2020), and holiday is 1 if a holiday, otherwise zero. The calendar indicates a few weekend days as workdays. The weekend and holiday variables were added to adjust for the likelihood that travel is different and workplace exposures are reduced on non-workday weekend days and holidays. Nanjing was included as 1 if Nanjing and 0 otherwise because that city had a substantially lower death rate per population unrelated to the predictor variables. “Deaths” is the death count for each day in each city during three years. The average deaths per day were 3.2 transport injuries and 4.4 other injuries. Log (population) was included as an offset variable to correct for differences in population size among the cities.

## Results

Table 1 presents the injury deaths per million inhabitants per year by external cause (transport vs. other) and in total for each city. In many instances, Nanjing’s rate is less than half that of 8 of the 9 other cities. The three year upward trend in total injury death rates in several cities is mainly due to unintentional injuries other than those experienced in transport.

**Table 1:** Annual Transport and Other Fatal Unintentional Injuries Per Million Inhabitants Among Cities in Jiangsu Province, China, 2015–2017

	Transport			Other Injury			Total		
	2015	2016	2017	2015	2016	2017	2015	2016	2017
Nanjing	81	79	80	120	147	138	202	226	218
Wuxi	115	115	112	273	315	314	387	430	426
Xuzhou	228	224	221	183	197	231	411	421	452
Changzhou	150	168	170	246	295	301	395	463	471
Nantong	187	214	232	274	365	380	461	579	612
Lianyungang	212	212	227	295	320	338	507	532	565
Huaian	210	196	189	218	241	244	428	437	433
Yancheng	206	218	261	199	289	282	404	508	543
Suqian	162	179	183	137	151	180	299	330	364

Totals may not add exactly because of rounding

Table 2 gives the means and standard deviations of the weather and pollution variables by city. Nanjing is not consistently higher or lower on the predictor variables. These findings led to the decision to include Nanjing as a separate variable in the analysis.

**Table 2:** Means and Standard Deviations of Transformed Predictor Variables

	Temperature (°C)	Precipitation (mm)	Ozone (μg/m^3^)
	Mean (SD)	Mean (SD)	Mean (SD)
Nanjing	21.2 (9.1)	1.3 (2.7)	4.6 (0.4)
Wuxi	21.5 (9.1)	0.4 (2.6)	4.6 (0.5)
Xuzhou	20.5 (9.6)	0.9 (1.9)	4.5 (0.4)
Changzhou	21.4 (9.2)	1.5 (2.6)	4.5 (0.5)
Nantong	20.9 (8.9)	1.4 (2.6)	4.5 (0.5)
Lianyungang	19.6 (9.4)	0.8 (1.9)	4.6 (0.4)
Huaian	20.3 (9.2)	1.1 (2.2)	4.6 (0.4)
Yancheng	20.0 (9.1)	1.2 (2.4)	4.6 (0.4)
Suqian	20.4 (9.3)	0.9 (2.1)	4.6 (0.3)

**Fig. 1:**
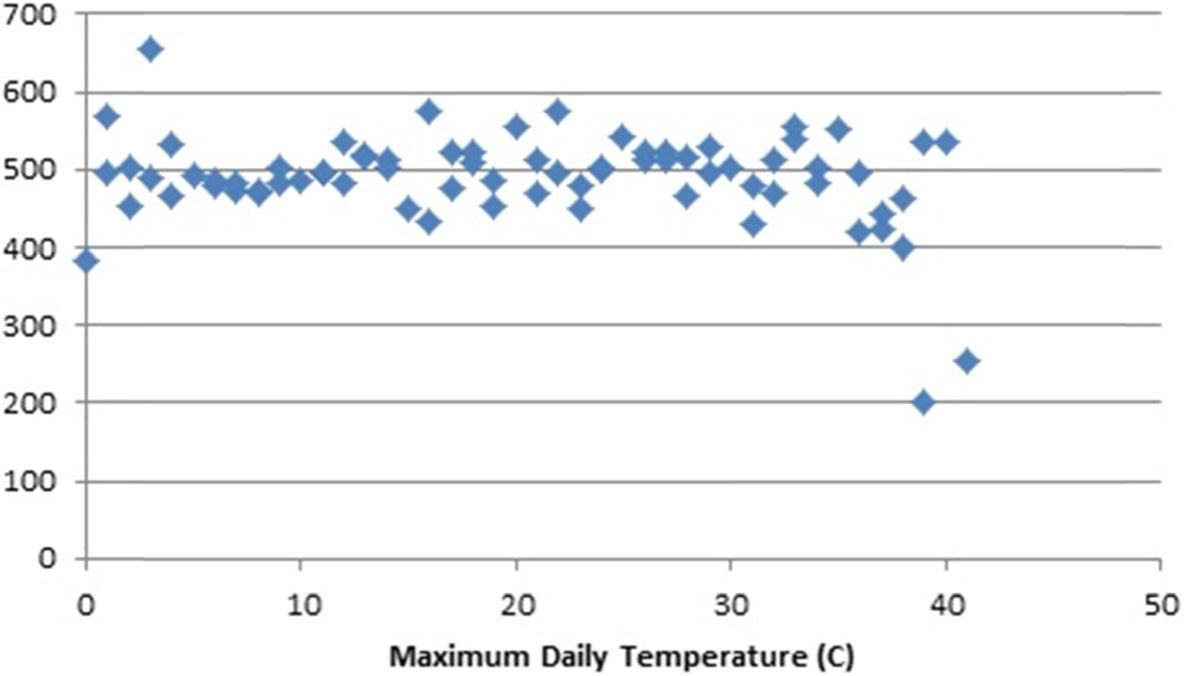
Maximum Daily Temperature (C) and Transport Deaths Per Billion Person Days of Exposure, Jiangsu Province, China, 2015–2017

**Fig. 2:**
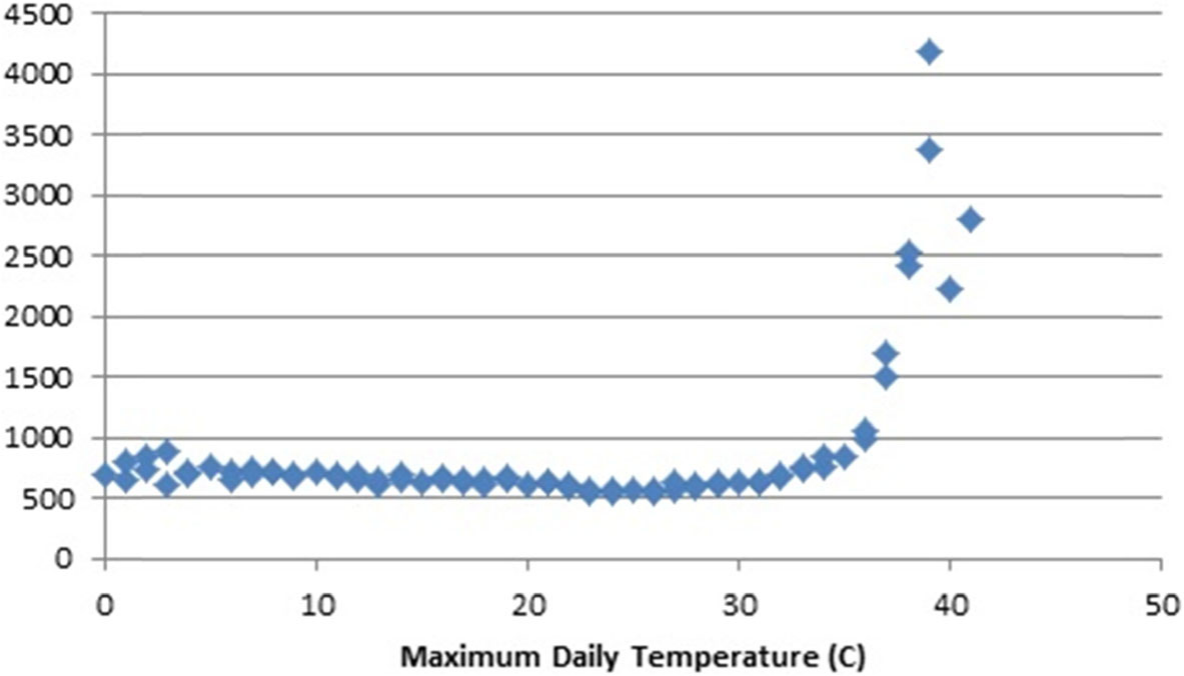
Maximum Daily Temperature (C) and Unintentional Injury Deaths Other Than Transport Per Billion Person Days of Exposure, Jiangsu Province, China, 2015–2017

Fig. 1 shows the transport related deaths per billion person days of exposure to specific maximum temperatures. Each data point is the sum of the deaths on the days that the maximum temperature reached the indicated degrees divided by the sum of the approximate number of people residing in a given city each day the temperature reached the indicated degrees adjusted to billions of person days. A maximum daily temperature at freezing or below in Jiangsu Province is rare. Person days when the maximum temperature was zero (C) or less were two-tenths of 1 % of the total so deaths on those days and the person years are included in the zero category in the graphs. There does not appear to an association of transport death risk with maximum temperatures other than a dip at the highest temperatures. In contrast, Fig. 2 indicates that unintentional injuries other than in transport declined slightly up to about 25 degrees maximum daily temperature, accelerated upward at higher temperatures and did so extraordinarily at temperatures above 34 degrees.

These findings led to the decision to analyze the data separately for cool (< 25 degrees), moderate (25–34 degrees) and hot (35 plus degrees) temperatures.

The Poisson regression coefficients are presented in Table 3 along with 95% confidence intervals and criteria for goodness of fit. Both transport and other fatal injuries are consistently lower for residents of Nanjing. Corrected for that and the estimated effects of other factors, transport deaths were related to increasing deaths when temperatures were low but the correlation reversed at higher temperatures. Non-transport injury deaths increased substantially in relation to higher temperatures when temperatures were in the moderate range and even more so at temperatures 35 degrees (C) and higher. Deaths were lower on rainy days when temperatures were cool and moderate with the exception of non-transport injuries when temperatures were moderate. Higher ozone concentrations were associated with more deaths except when temperatures were low. Transport fatalities were consistently lower on weekends and on holidays at high and low temperatures. Other injury deaths were higher on holidays when temperatures were low to moderate.

**Table 3:** Poisson Regression Coefficients beta and 95 Percent Confidence Intervals (CI): Unintentional Injury Fatalities Per Day

Transport Deaths						
Temperature	Cool (< 25 °C)		Moderate (25–34 °C)		Hot (> 34 °C)	
	Beta	95% CI	beta	95% CI	beta	95% CI
Temperature	−. 0077	(.0050, .0104)	−. 0074	(−.0145,−.0003)	−. 0796	(−.1202, −.0334)
√Precipitation	−.0189	(−.0260, −.0118)	−.0129	(−0.0209, −.0049)	−.0031	(−.0365, .0303)
Log (ozone)	−.1263	(−.1681, −.0845)	0.0563	(.0000, .1126)	−.1995	(−.0061, −.3929)
Weekend	−.0531	(−.0862, −.0120)	−.0534	(−.0959,−.0109)	−.1793	(−.3019, −.0567)
Holiday	−.0863	(−.1441, −.0285)	.0765	(.0094, .1435)	−.3979	(−.7640, −.0318)
Nanjing	−.8241	(−.8827, −.7655)	−.9118	(.9898, −.8338)	−.7803	(−.9695, −.5911)
Intercept	−13.9409		−14.4360		−12.6328	
Deviance/df	1.35		1.37		1.39	
Other Unintentional Injury Deaths						
Temperature	Cool (< 25 °C)		Moderate (25–34 °C)		Hot (> 34 °C)	
	beta	95% CI	beta	95% CI	beta	95% CI
Temperature	−.0045	(−.0068, −.0021)	.0314	(.0251, .0377)	.2277	(.2037, .2517)
√Precipitation	−.0170	(−.0233, −.0107)	.0095	(.0026, .0164)	.0101	(−.0313, .0111)
Log (ozone)	−.1586	(−.1945, −.1227)	.0248	(−.0248, .0744)	.1788	(.0536, .3040)
Weekend	−.0051	(−.0337, .0235)	−.0146	(−.0524, .0232)	−.0325	(−.1034, .0384)
Holiday	−.0269	(−.0211, −.0749)	.0208	(−.0415, .0831)	.2220	(−.4780, .0380)
Nanjing	−.6288	(−.6758, −.5817)	−.6376	(−.6996, −.5756)	−.6667	(−.7728, −.5606)
Intercept	−13.3868		− 15.2458		−22.6963	
Deviance/df	1.35		1.40		3.18	

The criteria for goodness of fit are near 1 indicating good fit of the models

Of concern in use of regression coefficients is the degree to which the predictor variables are correlated. The squared OLS correlation coefficients among the predictor variables are displayed in Table 4. None of the correlations are high enough to be of concern. O_3_ is uncorrelated with precipitation and is only moderately correlated with temperature at cool temperatures and less so at moderate and high temperatures in this study. The correlation of these variables with weekends, Nanjing and population was less than 0.01. Nanjing’s larger population resulted in an R^2^ of 0.22 with population.

**Table 4:** Squared OLS Correlation Coefficients Among Transformed Daily Weather and Pollution Predictor Variables At Cool, Moderate and Hot Temperatures, Jiangsu Province, China. 2015–2017

	Cool		Moderate		Hot	
	Precipitation	Ozone	Precipitation	Ozone	Precipitation	Ozone
Temperature	0.04	0.21	< 0.01	0.01	< 0.01	0.04
Precipitation		< 0.0		0.11		0.02

## Discussion

The differences in association of temperature, precipitation, pollution and fatal injury depending on temperature range is evidence of what is called “effect modification”, also found in research on non-injury mortality in relation to temperature and air pollutants (Chen et al. 2018). These results are mostly consistent with previous studies showing increased risk of unintentional injury mortality as temperatures rise, particularly when maximum daily temperatures are above 25 degrees (C). A major exception in Jiangsu Province is that the risk of death in relation to rising temperatures is mainly confined to people engaged in activities other than transport. While the results are correlations with the usual concern that they may not represent causation, the cited literature in the introduction suggests that people adjust their activities to pollution and it is common knowledge that they do so in response to weather.

The results are unlikely to be the result of confounding by unmeasured variables. Confounding occurs when an unmeasured variable causes both predictor and outcome variables resulting in their being correlated without one causing the other. The causes of variations in temperature and precipitation are unlikely to influence mortality risk. Workers in industries that increase pollution may be at greater risk of fatal injury but the correlation of increases in ozone to injury is negative at low temperatures.

A major limitation of this study is lack of observations of actual behavior under the conditions correlated to mortality. More research is needed on observed behavior in relation to temperature, precipitation and concentration of pollutants. What are people doing that exposes them to the energy (or lack thereof in the cases of drowning and asphyxiation) at varying temperatures, on rainy days or when pollution is higher? Why would warmer temperatures be related to transport casualties in Spain and fatalities in U.S. cities much more strongly than in cities in Jiangsu Province, China? One possible reason is the difference in cultures regarding vehicle use. U.S. road deaths are substantially higher on Friday and weekend days than on other weekdays (Bureau of Transportation Statistics 2019). In contrast, transport deaths in Jiangsu Province are 3 % less on Saturday and 6 % less on Sunday than on the average weekday. This suggests that vehicles are used less for discretionary travel in Jiangsu Province. Also, a substantial proportion of the correlation of temperature and road death risk in the U.S. is due to the apparent lack of use of bicycles and motorcycles at subfreezing temperatures (Robertson 2018b). Such temperatures are extremely rare during daytime in Jiangsu Province.

Unfortunately, we have no data on kilometers travelled per vehicle in China or pedestrian volume and bicycle use in the U.S. and Chinese cities. If there were major road injury prevention efforts during 2015–2017 in Jiangsu Province, they could have offset the effect of temperature. According to one report, China has made little use of “traffic calming” road designs that have reduced fatality rates in other countries but does have engineering standards for vehicle crashworthiness, child safety seats and school bus seats (Fayard 2017). A seat belt use law was imposed throughout China in May, 2004. A study if belt use observed in traffic in Nanjing soon after the law indicated about 67% use by drivers and 19% by front seat passengers (Routley et al. 2007). During 2005–2007, belt use by drivers in Nanjing had declined to 39% and that of front seat passengers to 3% leaving substantial room for improvement (Routley et al. 2008). A study of road deaths in Jiangsu Province during 2012 found that more than half the deaths occurred to pedestrians and that the risk of pedestrian deaths per population increased dramatically with age (Ding et al. 2017) but the age distribution of the population would not have changed enough in three years to affect the results reported here. A search of Chinese laws and regulations aimed at childhood injury prevention relative to laws and regulations recommended by the United Nations Children’s Fund, the World Health Organization or the European Child Safety Alliance found that 10 of 27 were not found in Chinese statutes and regulations (Li et al. 2015).

The results regarding the possible effects of ozone concentrations suggest that avoidance behavior may reduce injury risk at low temperatures but the net effect of more pollution during days of moderate and high temperatures is increased injury risk. The lower deaths on rainy days are suggestive of avoidance behavior.

We have no data that informs the reasons for the lower death rates in Nanjing. Study of the differences in local injury prevention efforts in that city compared to the others might reveal measures that could be applied elsewhere.

## Conclusions

The association of warming and injury mortality varies from country to country depending on the temperature ranges experienced, cultural factors and behavioral responses to temperature, precipitation and air pollution. With a few exceptional circumstances, warming temperatures are associated with increased risk of unintentional injury mortality. Without further efforts at prevention based on data regarding the types and circumstances of injuries affected, climate warming will likely contribute to an upward trend in injury mortality. Burning fossil fuels that contribute to warming and air pollution continues to grow, outpacing the adoption of sustainable energy sources (United Nations Environment Programme 2019).

## Data Availability

Daily mortality data is not available for public access.
